# Promoter hypermethylation of FANCF and outcome in advanced ovarian cancer

**DOI:** 10.1038/sj.bjc.6604325

**Published:** 2008-04-15

**Authors:** S L Lim, P Smith, N Syed, C Coens, H Wong, M van der Burg, P Szlosarek, T Crook, J A Green

**Affiliations:** 1 Division of Surgery and Oncology, School of Cancer Studies, University of Liverpool, Liverpool, UK; 2 The Breakthrough Toby Robins Breast Cancer Research Centre, Chester Beatty Laboratories, The Institute of Cancer Research, London, UK; 3 EORTC Data Center, Brussels, Belgium; 4 Clatterbridge Centre for Oncology NHS Trust, Wirral Merseyside, UK; 5 Erasmus University Medical Center, Rotterdam, The Netherlands; 6 Lung and Mesothelioma Unit, Department of Medical Oncology, St Bartholomew's Hospital, London, UK

**Keywords:** ovarian cancer, Fanconi genes, platinum resistance, clinical trial

## Abstract

The Fanconi gene family has a role in DNA repair and inactivation of FANCF has been proposed as a mechanism of sensitisation to platinum chemotherapy. This study sought to confirm this hypothesis in cell lines and a large series of ovarian cancer samples. Promoter methylation was assessed by methylation-sensitive polymerase chain reaction of FANCF in nine ovarian cancer cell lines and 74 ovarian cancer samples taken from patients entered on a trial of cisplatin-based chemotherapy. This study confirmed methylation-dependent silencing of FANCF in one out of nine ovarian cancer cell lines. Methylation of FANCF was demonstrated in 13.2% of 53 evaluable ovarian tumour samples. Progression-free survival gave an HR of 3.63 (95% CI: 1.54–8.54, *P*=0.0016) in favour of the unmethylated cases. There was no association with overall survival. This study does not support methylation-dependent silencing of FANCF as a mechanism of sensitisation to platinum-based chemotherapy in ovarian cancer.

Ovarian cancer is in many cases a chronic disease characterised by protracted sensitivity to anticancer agents, of which the most active are the platinum coordination complexes. These are thought to lead to cell death through DNA cross-linking, causing unrepairable DNA damage and activation of apoptosis. While the majority of cases initially respond to the platinum compounds, resistance eventually develops by mechanisms that remain poorly defined (Shah and Schwartz, 2001). The Fanconi DNA repair pathway protects cells against death and genotoxicity induced by cross-linking agents, including cisplatin and the alkylating agents. One function of the FANC proteins is to support the integrity of the RAD51-, NBS1-, BRCA1- and BRCA2-dependent pathways for DNA repair, and also suppressing apoptotic responses to nonchemical extracellular signals (Bagby and Olson, 2003). Defects in these proteins are associated with Fanconi anaemia, a syndrome characterised by hypersensitivity to DNA cross-linking agents (D'Andrea and Grompe, 2003). At least seven genes encoding proteins participating in the Fanconi pathway have been identified. These encode proteins that cooperate in a DNA damage response, including FANCD1 (BRCA2).

Methylation-dependent transcriptional silencing of FANCF occurs in cisplatin-sensitive epithelial ovarian cancer cell lines, implying that platinum resistance correlates with the methylation state of the CpG island of FANCF and suggesting a model of reversible methylation of FANCF leading initially to chromosomal instability associated with platinum sensitivity, followed by demethylation and expansion of platinum-resistant clones (Taniguchi *et al*, 2003). These authors found no mutation in the FANCF gene, but methylation of the promoter region of FANCF in 4 out of 19 (21%) ovarian cancer samples. This finding was subsequently confirmed by Wang *et al* (2006), who found methylation in 5 out of 18 cases of ovarian cancer, but no study has formally addressed the issue of whether loss of FANCF function, via methylation-dependent silencing, influences clinical outcome in patients treated with cisplatin. Here, we have analysed the methylation status of the FANCF CpG island in a series of ovarian cancer patients treated on a clinical trial to address the possibility that methylation of FANCF is a determinant of clinical response and outcome in ovarian cancer patients treated with cisplatin-based chemotherapy.

## MATERIALS AND METHODS

### Cell lines

The following epithelial ovarian cancer cell lines were used: 1847, SKOV3, TR175, JAMA2, OVCAR3 and OVCA433. A2780 and its cisplatin- and doxorubicin-resistant derivatives, A2780cis and A2780adr, respectively, were obtained from the European Collection of Cell Cultures (ECACC). Cells were routinely grown in DMEM supplemented with 10% fetal bovine serum. Drug resistance of A2780cis and A2780adr was maintained by exposure to each agent as recommended by the supplier. Primary, ovarian germinal epithelial cells were prepared and cultured as described by Kruk *et al* (1990). Genomic DNA was prepared from exponentially growing cells using proteinase K digestion and total RNA using RNAzol B.

### Ovarian cancer tissue samples

A single representative tissue block was obtained from 74 patients entered on a randomised controlled trial carried out by the EORTC (Study 55931). This randomised 640 patients with predominantly stage III and IV disease to cisplatin (80 mg m^−2^) and cyclosphosphamide (800 mg m^−2^) (CP) or cisplatin (80 mg m^−2^) and paclitaxel (175 mg m^−2^) (TP) over 3 h for six cycles (Piccart *et al*, 2003). At a median follow-up of 6.5 years, this study showed a 4-month improvement in survival in favour of the cisplatin/paclitaxel arm (HR=0.75, 95% CI: 0.63–0.90, *P*=0.001). This translational study was approved by the Wirral Research Ethics Committee, UK. Ten-micron sections were cut for DNA extraction and adjacent four-micron sections stained by H&E and immunohistochemistry. Genomic DNA was extracted from the single 10-*μ*m tissue section using the Pinpoint DNA Isolation System TM (Zymo Research, Cambridge, UK).

### Analysis of FANCF expression and methylation

For analysis of FANCF mRNA expression in ovarian cancer cell lines, 1 *μ*g total RNA was converted to first-strand cDNA using M-MLV Reverse Transcriptase and the Oligo (dT)12–18 primer (Invitrogen, Paisley, UK). Reverse transcription-polymerase chain reaction (RT-PCR) primers were as follows: FANCF forward, 5′-AGGAGGACTCTCTGATGAAGACCC-3′ and reverse, 5′-CAGGTGATTTGTGGATGCCG-3′; and GAPDH forward, 5′-TGAAGGTCGGAGTCAACGGATTG-3′ and reverse, 5′-GCCATGAATTTGCCATGCCATGGGTGG-3′.

Polymerase chain reaction was for 26 cycles for both FANCF and GAPDH, after which reaction products were resolved on 2% agarose gels.

Methylation in FANCF was analysed using methylation-sensitive PCR (MSP) (Herman *et al*, 1996), using the primers and conditions described by Taniguchi *et al* (2003). Briefly, 1 *μ*g of genomic DNA was subjected to sodium bisulphite conversion using EZ DNA Methylation Kit (Zymo Research). Control genomic DNAs were (i) methylated human genomic DNA (Chemicon®, Hampshire, UK) and (ii) DNA isolated from the peripheral blood lymphocytes of healthy individuals. Methylation-sensitive PCR was performed in a reaction volume of 20 *μ*l for 38 cycles after which products were resolved on 2% agarose gels and visualised under UV illumination to compare unmethylated and methylated amplifications. Samples were scored by visual comparison of unmethylated and methylated reactions.

Each DNA was analysed a minimum of three times. Samples were deemed positive if FANCF MSP primers were as follows: methylated forward, 5′-TTTTTGCGTTTGGAGAATCGGGTTTTC-3′ and reverse, 5′-ATACACCGCAAACCGCCGACGAACAAAACG-3′; unmethylated forward, 5′-TTTTTGTGTTTGTTGGAGAATTGGGTTTTT-3′ and reverse, 5′-ATACACCACAAACCACCAACAAACAAAACA-3′.

Bisulphite sequencing was carried out using the following primers: forward, 5′-TTTTTGTTTTTATTGGTTGTGTAGT-3′ and reverse, 5′-AAATCCCTTCTACAACACCTAAATC-3′.

Polymerase chain reaction products were purified with a PCR Purification Kit (Qiagen Ltd, West Sussex, UK), ligated into a TA cloning vector (Invitrogen Ltd) and transformed into Top 10 *Escherichia coli* competent cells (Invitrogen Ltd). Colonies were grown on LB-agar plates under ampicillin and blue/white selection. Insert-containing colonies were cultured and plasmid DNA isolated and used as template in a dideoxy cycle sequencing reaction using the Big Dye Terminator Cycle Kit (PE Applied Biosystems, Foster City, CA, USA) and reverse primers. For each sample, a minimum of 12 clones were sequenced, to determine the overall level of methylation within CpG islands.

### Statistical analysis

Overall survival (OS) time and disease-free survival time were defined as the period that elapsed from primary surgery to death and to death or relapse, respectively. Kaplan–Meier analyses and the log-rank test (Kaplan and Meier, 1958; Tarone and Ware, 1977) were used to estimate and compare OS and disease-free survival curves. The independent effects of prognostic factors and other covariates on survival function were determined by the COX proportional-hazards regression model, stratified for the assigned treatment group (Cox, 1972). Correlations between various factors were assessed by Spearman's rank correlations. The proportionality assumptions of the method were tested graphically by looking at the log-minus-log survival function plots. The stability of the variables entered in the final model was examined by repeating the analysis excluding each factor in turn.

## RESULTS

### FANCF methylation in epithelial ovarian cancer cell lines

**Figure 1:**
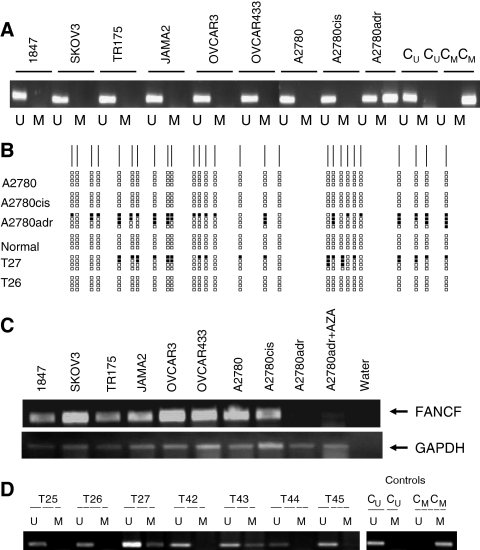
Methylation analysis of the FANCF CpG island in epithelial ovarian cancer. (**A**) Methylation-sensitive PCR analysis of the FANCF CpG island in ovarian cancer cell lines. Methylation-sensitive PCR was performed in the indicated cell lines as described in the Materials and Methods. For each cell line, unmethylated (U) and methylated (M) reactions are shown. Control unmethylated (C_U_) and methylated (C_M_) reactions are also shown. (**B**) Bisulphite sequencing of part of the FANCF CpG island. The CpG sites are shown as vertical lines on the top horizontal line. Methylated CpG sites are shown as black blocks. Five levels of methylation are indicated: 0 – no black blocks; 1–25% – one black block; 25–50% – two black blocks; 50–75% – three black blocks; and 75–100% – four black blocks. (**C**) Reverse transcription-polymerase chain reaction analysis of FANCF expression in epithelial ovarian cancer cell lines. Reverse transcription-polymerase chain reaction was performed in the indicated cell lines as described in the Materials and Methods. In the case of A2780adr, reactivation of expression by AZA is also shown. (**D**) Methylation analysis of the FANCF CpG island in primary ovarian carcinomas. Methylation-sensitive PCR was performed in the indicated cell lines as described in the Materials and Methods. Methylation is readily detectable in cases T27 and T43. Control unmethylated (C_U_) and methylated (C_M_) reactions are also shown.

We analysed methylation in the FANCF CpG island in a panel of ovarian cancer cell lines. We detected no evidence of methylation in 1847, SKOV3, TR175, JAMA2, OVCAR3, OVCA433, A2780 and its cisplatin-resistant derivative A2780cis. However, methylation was clearly and reproducibly detected in the doxorubicin-resistant derivative A2780adr, while methylated and unmethylated controls showed only appropriate amplified products (Figure 1A). To confirm these results, we performed bisulphite sequencing of the FANCF CpG island in A2780, A2780cis, A2780adr and in normal ovarian germinal epithelium. These studies verified that methylation was not detectable in A2780, A2780cis or normal ovarian epithelium, but was clearly present in A2780adr (Figure 1B). By semiquantitative RT-PCR, FANCF mRNA was readily detected in each of the cell lines, but was expressed at a very low level in A2780adr, consistent with the presence of methylation (Figure 1C). To verify that transcriptional downregulation was associated with aberrant CpG methylation, A2780adr cells were treated with the demethylating agent 5-azacytidine (AZA). The steady-state level of FANCF mRNA was clearly upregulated by exposure to this agent (Figure 1C), consistent with the hypothesis that aberrant methylation is the mechanistic basis for downregulation of the mRNA. In additional analyses, we did not observe transcriptional downregulation of FANCA, FANCC, FANCD1, FANCD2, FANCE or FANCG mRNA and found no evidence of methylation in the CpG islands of any of these genes in our ovarian cancer cell line panel. These results reveal downregulation of FANCF mRNA in the doxorubicin-resistant A2780adr epithelial ovarian carcinoma cell line and show that this correlates with aberrant CpG island methylation.

### Ovarian cancers

**Figure 2:**
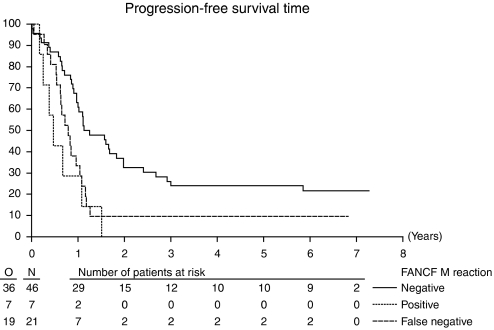
Kaplan–Meier curves for survival for 74 ovarian cancer patients by FANCF methylation status (*P*=0.0016). Uninformative cases shown as false negative cases (dotted line). Methylation-positive cases are shown by the continuous line, and negative cases by the dashed line.

Using the same MSP primers, we next analysed methylation in the FANCF CpG island (Figure 2) in the 74 patients. We reproducibly detected methylation in seven (9.4%) cases. To confirm the sensitivity and specificity of the MSP reaction conditions, we performed bisulphite sequence analysis of cases classified as positive or negative by MSP. There was no evidence of methylated CpGs by bisulphite sequencing in case 26 (unmethylated by MSP), but clear evidence in case 27 (methylated by MSP) (Figure 1C).

To assess whether the 74 patients in this study were representative of the trial population, the patient characteristics were compared with the other 317 patients randomised by EORTC and NCIC centres, which contributed to the sample collection, and no significant differences were observed. The median survival of the 74 patients was 2.31 years (95% CI: 2.65–3.25) and 2.57 years (95% CI: 2.17–2.97) in the other 317 randomised patients. Of the 67 FANCF patients negative for FANCF methylation, 31 were in the CP and 36 in the TP arm. Of the seven positive patients, four were on the CP arm and three received TP.

Table 1 shows the correlation with clinicopathological variables in the series. Methylation of FANCF was seen more commonly in 85.7% of the serous subtypes compared with 65.7% of the nonserous tumours but this was not significant (*P*=0.32). There was no association with age, stage, grade or residual tumour mass. Methylation of FANCF was not observed in any of six normal ovarian germinal epithelial preparations.

Analysis of response and FANCF status is limited by the small numbers, and the number of categories requiring grouping of CR (complete response), PR (partial response) SD (static disease), NED (no evidence of disease after primary surgery), PD (progressive disease) and NE (not evaluable). Across the whole group, there was no association between response and FANCF status (*P*=0.331). In the 34 patients on the cisplatin/cyclophosphamide arm, methylation-negative status was associated with the CR+PR+SD group compared with the PD category (*P*=0.0098) after exclusion of the nonevaluable cases (Table 2). No such association was seen in the cisplatin/paclitaxel arm.

The analysis of survival outcome was performed excluding the 21 cases negative in both the U and M reactions, as these are regarded as uninformative. On this basis, the proportion of cases positive for methylation of FANCF becomes 13.4%. Progression-free survival (PFS) gave an HR of 3.63 (95% CI: 1.54–8.54, *P*=0.0016) in favour of the unmethylated cases as shown in Figure 2. When adjusted for treatment, stage, residual mass after surgery, WHO performance status and age in multivariate analysis, the HR was 3.7 (95% CI: 1.43–9.54, *P*=0.001). These data are consistent with an adverse outcome for methylated cases when treated by platinum-based chemotherapy. For the analysis of OS, the HR was 1.56 for FANCF-negative status (95% CI: 0.65–3.74, *P*=0.31). These values are not significant. When adjusted for the same factors, the HR for OS fell to 1.162 (95% CI: 0.434–3.113, *P*=0.77). There was no interaction between treatment arm and either PFS or OS. Response was not associated with survival.

Analysis of the PFS at greater or less than 10.5 months, a time point chosen as treatment time on platinum-based chemotherapy plus 6 months, to correspond with the generally accepted time point to separate platinum-refractory/resistant patients from sensitive patients Markman and Bookman (2000), shows that FANCF methylated patients are 2.5 times more likely to have progressed than FANCF unmethylated cases.

## DISCUSSION

In this study, we have addressed the hypothesis that methylation-dependent transcriptional silencing of FANCF is a determinant of clinical response and outcome to platinum-based adjuvant chemotherapy of epithelial ovarian cancer.

The original observations of Taniguchi *et al* (2003) suggested that reduced expression of FANCF would be associated with increased sensitivity to cisplatin based on studies on two cell lines and a small number of clinical samples whose treatment or outcome were not specified. In the present study, we have observed FANCF methylation in one cell line, but failed to show methylation by MSP and bisulphite sequencing in either the cisplatin-sensitive A2780 or its derivative with acquired cisplatin resistance, A2780cis. In contrast, FANCF mRNA levels were greatly reduced in the doxorubicin-resistant A2780adr and this correlated with aberrant methylation in the FANCF CpG island, as assessed by MSP and bisulphite sequencing. The mechanistic association between methylation and transcriptional downregulation was supported by partial reactivation by AZA pretreatment, consistent with an epigenetic mechanistic basis for silencing of the gene.

Our detection of methylation in the FANCF CpG island (9.4%) is comparable to the frequencies reported by Taniguchi *et al* (2003) and Wang *et al* (2006). A fourth study by Teodoridis *et al* (2005) failed to detect FANCF methylation in 106 stage III and IV ovarian cancers. It is unclear whether the differences between the studies that are positive for methylation (including the present one) and that of Teodoridis are due to differences in techniques or patient populations. It should be noted that cases positive for methylation by MSP in our series of patients were confirmed by bisulphite sequencing, both in the A2780adr cell line and in primary ovarian cancers. Furthermore, our data are broadly consistent with studies of other cancer types. For example, methylation of FANCF has been reported in 6.7% (4/60) testicular tumours Koul *et al* (2004), 15% (13/89) head and neck cancers (Marsit *et al*, 2004) and 30% (30/100) cervical cancers (Narayan *et al*, 2004). Moreover, in a study of 158 cases of nonsmall cell lung cancer, Marsit *et al* (2004) found 22 (14%) to have promoter methylation of FANCF, and this was also associated with adverse survival (HR=3.1, 95% CI: 1.2–7.9, *P* not reported).

In this series of patients treated on a clinical trial with standardised treatment and follow-up, FANCF methylation was an adverse prognostic factor, at least for PFS, and there was no significant effect on survival. One arm was treated with cisplatin and cyclophosphamide, both cross-linking agents, while the other arm was treated with cisplatin plus a taxane, and even in the former group treated with two cross-linking agents, after allowing for the small numbers, the response data provide no evidence in favour of FANCF methylation-sensitising patients to this chemotherapy combination. It is therefore likely that FANCF methylation is not a major feature of chemonaive ovarian cancers, but may be a ‘progression factor’ occurring late in the process of carcinogenesis. Furthermore, methylation of FANCF is unlikely to have a major impact on response to established anticancer agents in epithelial ovarian cancer.

One explanation of the disparity may lie in the relationship between genotoxicity and apoptotic cell death. A cell may suffer major genotoxic damage, which does not lead to apoptosis as there are different pathways involved in these processes (Bagby and Olson, 2003). The overall chemosensitivity effect is likely to be due to the interaction of multiple pathways subject to control by genetic and epigenetic mechanisms. The extent to which these pathways are linked is unknown in human tumours, and the therapeutic implications are complex. For example, if the Taniguchi model were correct, the predicted effect of the use of nonselective demethylating agents would be to reactivate FANCF expression, and thereby increase cellular resistance to cisplatin. Conversely, Gifford *et al* (2004) have clearly shown that acquired methylation of hMLH1 is associated with clinical resistance to cisplatin-based chemotherapy.

The inactivation of FANCF as a marker of platinum sensitivity may be too simplistic, as Taniguchi *et al* concede in their description of sequential changes in methylation status during the life cycle of the tumours. It should also be pointed out that the samples in this study were taken prior to cisplatin exposure, and the methylation status of FANCF may have changed in some patients after exposure. Repeat biopsy at relapse or progression would be necessary to confirm this and determine the dynamics of methylation, drug response and outcome. Alternatively, it may be possible to detect methylated FANCF DNA in peripheral blood to address this.

## Figures and Tables

**Table 1:** Relation of FANCF methylation status and clinicopathologic variables in 54 cases of advanced ovarian cancer

	**FANCF methylated, *n*=7 (column %)**	**FANCF unmethylated, *N*=46 (column %)**	**Pearson's *χ*^2^/Fisher's exact *P***
Median age (range, years)	64.1 (42.3–65.9)	59.3 (22.8–76.4)	0.937^*^
Age <65	3 (42.9%)	36 (53.7%)	0.701
Age >65	4 (57.1%)	31 (46.3%)	
			
*Performance status*			
0	2 (28.6%)	22 (47.8%)	0.688
1	3 (42.9%)	15 (32.6%)	
2	2 (28.6%)	8 (17.4%)	
3	0 (0%)	1 (2.2%)	
			
*Stage*			
IIB/IIC/III	5 (71.4%)	36 (78.3%	0.462
IV	2 (28.6%)	9 (19.6%)	
Missing	0 (0%)	1 (2.1%)	
			
*Histology*			
Serous	6 (85.27%)	27(58.7%)	0.136
Nonserous	0 (0%)	12 (26.1%)	
Missing	1 (14.3%)	7 (15.2%)	
			
*Tumour grade*			
Well-moderate	3 (42.9%)	21 (45.7%)	0.449
Poorly differentiated	4 (57.1%)	18 (39.1%)	
Missing	0 (0%)	87 (15.2%)	
			
*Residual tumour volume*			
<1 cm	1 (14.3%)	13 (28.3%)	0.383
⩾1 cm	6 (85.7%)	32 (69.6%)	
Missing	0 (0%)	1 (2.1%)	

^*^Mann–Whitney *U*-test (two-sided) *P*-value.

**Table 2:** Response in 74 patients at the end of treatment (surgery and chemotherapy) by FANCF status (excludes 21 uninformative patients)

	**Cisplatin+cyclophosphamide^a^**		**Cisplatin+paclitaxel**	
**Response (WHO)**	**FANCF−**	**FANCF+**	**FANCF−**	**FANCF+**
CR	8 (26.7)	0	7 (18.9)	1 (33.3)
PR	5 (26.7)	1 (25)	4 (10.8)	0
SD	1 (3.3)	0	2 (5.4)	1 (33.3)
NED	3 (10)	0	10 (27.0)	0
PD	2 (6.7)	3 (75)	3 (8.1)	0
Missing	1 (3.3)	0	0	0
NE	10 (33.3)	0	11 (29.7)	1 (33.3)

CR=complete response; NE=not evaluable; NED=no evaluable disease; PD=progressive disease; PR=partial response; SD=static disease.

a Association between negative FANCF status and response (CR+PR+SD *vs* PD, excluding NE and NED) *P*=0.0098. There was no correlation between response and methylation status in the cisplatin and paclitaxel group (*P*=0.5023).
